# Antiphospholipid (Hughes) syndrome: beyond pregnancy morbidity and thrombosis

**DOI:** 10.1186/1740-2557-6-3

**Published:** 2009-05-19

**Authors:** Maria Mialdea, Shirish R Sangle, David P D'Cruz

**Affiliations:** 1 The Lupus Research Unit, The Rayne Institute, 4thFloor, Lambeth Wing, St Thomas' Hospital, London, SE1 7EH, UK

## Abstract

The antiphospholipid syndrome is an autoimmune disease characterised by recurrent arterial or venous thrombosis, pregnancy morbidity and the persistence of positive antiphospholipid antibodies. Many other clinical manifestations may occur including heart valve disease, livedo reticularis, thrombocytopenia and neurological manifestations such as migraine and seizures. We review a number of other manfestations including stenotic lesions, coronary artery disease and accelerated atherosclerosis, skeletal disorders and the concept of seronegative antiphospholipid syndrome.

## Introduction

The antiphospholipid (Hughes) syndrome (APS), first described in 1983, is an autoimmune disease characterised by recurrent arterial or venous thrombosis, pregnancy morbidity and the persistence of positive antiphospholipid antibodies (aPL) [1]. Although only thrombosis and pregnancy loss are included in the revised classification criteria for APS [2], other features are also described [3]. These include heart valve disease, livedo reticularis, thrombocytopenia and neurological manifestations such as migraine and seizures. Recently a number of other features have been described in APS, which we discuss in this review.

## Stenotic lesions and vasculopathy

Vascular occlusions are increasingly being recognized in patients with APS, although the exact etiology remains unclear [4]. We found a high prevalence of renal artery stenosis (RAS) in APS patients with uncontrolled hypertension compared to two control groups [5]. Other stenotic lesions such as coeliac [6] and intracerebral arterial stenosis [7] have also been reported. These stenotic lesions are smooth and well defined and are different from those seen in atherosclerotic RAS or fibromuscular dysplasia [5]. Interestingly histological examination in SLE patients with APS [8] and a case report of a resected superior mesenteric artery showed fibro-elastic thickening of the intima and thrombosis [9]. These findings suggest that thrombosis and intimal and smooth muscle hyperplasia may be responsible for the vasculopathy in APS. Preliminary reports suggest that anticoagulation with a high intensity international normalized ratio (INR) helps to control blood pressure and prevents re-stenosis in APS patients with RAS [10]. Similarly, other case reports emphasize the importance of high intensity INR in various stenotic lesions in APS patients [7,11,12].

## Coronary artery disease

Since the description of the APS syndrome, a number of cardiac manifestations have been described including cardiac valvular abnormalities (Libman-Sacks endocarditis) [13,14]. Coronary artery disease in young adults and coronary artery bypass graft occlusions have been reported in APS patients [15]. Although typical myocardial infarction (MI) is well described in patients with APS [16,17], a number of reports have described MI and so called Syndrome X in the absence of atherosclerotic obstructive coronary artery lesions [18-20]. Cardiac Syndrome X is defined by the presence of angina-like chest pain, a positive response to stress testing and normal coronary arteriograms. Syndrome X is seen in menopausal women [21] and so was linked to low oestrogen levels [22]. However, in APS patients, Syndrome X and MI were observed in young women who were not menopausal [23]. Histopathological findings in myocardial tissue of a patient with APS, showed a non-inflammatory micro-vasculopathy, characterized by thrombi, and further ultra-structural studies confirmed the thrombosis and demonstrated endothelial activation [24]. These findings support the hypothesis that the endothelial dysfunction and subsequent thrombosis seen in the APS patients may be responsible for Syndrome X/MI and argues against the lack of oestrogen theory. Experts in this field recommend long term anticoagulation in this group of patients [19,20].

## Cerebral manifestations

Although stroke is the only accepted neurological criterion for the diagnosis of APS, a number of other manifestations are observed in the APS. The spectrum of non- thrombotic cerebral manifestations may range from focal neurological lesions to diffuse global dysfunction. It includes severe headaches, often migranous, hemiplegic migraine, cognitive dysfunction and memory deficits, dysphasia (mixing or inappropriate words), behavioural changes and seizure disorders [25]. Extrapyramidal symptoms such as chorea have also been described in association with sub-cortical dementia in patients with APS [26]. Tektonidou et al noted a significant association between cognitive dysfunction and white matter lesions in the brain in patients with APS [27]. It is not uncommon to see white matter changes in the brain mimicking multiple sclerosis. Although a double blind cross-over trial comparing low molecular weight heparin with placebo failed to show positive results [28], clinical experience suggests that severe cognitive dysfunction and intractable headaches often respond to anticoagulation therapy in these patients [25].

## Skeletal manifestations of the APS

APS may involve multiple organs such as kidney, brain, eye, ear and liver and it may also affect the skeleton. A prospective cohort study [29] together with several case reports of osteonecrosis in primary APS in the absence of osteoporosis [30,31], have strengthened the possible association between aPL and osteonecrosis. Our own experience is that non-traumatic metatarsal fractures are more prevalent in APS/aPL positive patients [32,33]. Interestingly most had normal DEXA scans, none had any preceding trauma and none had received high doses of steroids. To assess the true prevalence of these fractures and their relation to aPL, a prospective study is needed in both symptomatic and asymptomatic patients.

## Endothelial dysfunction

Accelerated atheroma has been described as a feature of APS as in other autoimmune inflammatory conditions. APS/aPL are associated with accelerated atherosclerosis [34] by targeting some of the steps that constitute early atherogenesis from endothelial activation to oxidized LDL uptake by foam macrophages [35,36]. The ankle-brachial index (ABI) was found to be abnormal in patients with APS with thrombosis as well as in APS patients with pregnancy morbidity without thrombosis [37,38]. A recent study by Charakida et al of endothelial assessment in patients with APS is worth mentioning. She studied 90 age, sex and cardiovascular risk factor profile matched patients with primary APS and a control group of 90 people with negative aPL. High resolution ultrasound was used to determine carotid intima media thickness (cIMT), endothelium dependent flow mediated dilatation (FMD) and endothelium independent nitroglycerine mediated dilatation of the brachial artery. This data showed significantly reduced FMD and increased cIMT in APS patients as compared to healthy individuals [39]. This corroborates previous observations by Medina et al [40]. Similarly, observations by Ames et al of subclinical atherosclerosis using intima-medial thickness in patients with primary APS in their 4^th ^decade are worth mentioning [41]. This data indicates that endothelial dysfunction and pre-clinical atherosclerosis is prevalent in APS/aPL patients. The increased prevalence of an abnormal ABI in patients with APS, suggests that a large vessel vasculopathy could be a contributing factor to both thrombosis and pregnancy loss in APS [37,38].

## Complications following renal biopsy in patients with APS/aPL

Although APS is by definition a hypercoaguable state, a surprising recent preliminary report by Chaib et al found an increased risk of bleeding complications following renal biopsy in patients with lupus nephritis (LN) and APS/aPL as compared to LN alone [42]. This single centre study examined > 200 patients of which 86 were APS/aPL positive. The study identified a positive lupus anticoagulant and elevated serum creatinine levels as significant risk factors for post biopsy bleeding complications.

## Livedo reticularis and "Seronegative APS"

"Sero-negative" APS has remained an enigma and the concept is controversial. According to classification criteria for APS, aPL (lupus anticoagulant and anticardiolipin antibodies) and Beta 2 Glycoprotein I (B2GPI) antibodies are essential for the classification of patients with APS. Although aPL and anti-B2GPI are sensitive tests, they are not sensitive enough to pick up all patients with APS. A small group of APS patients remain persistently negative for routine assays of aPL [43,44]. Livedo reticularis was included in the original clinical description of the APS. Frances et al reported significant associations between pathological livedo reticularis (racemosa) and cerebral or ocular ischemic arterial events, seizures, heart valve abnormalities, hypertension and Raynaud's phenomenon in patients with APS [45]. As with APS, livedo reticularis in the absence of aPL has been associated with pregnancy morbidity and abnormal ABI [46]. Livedo reticularis shares a number of features with APS such as pregnancy loss, arterial thrombosis, heart valve abnormalities and seizures [47] and indeed it is the most common cutaneous manifestation of APS [48,49]. There is therefore increasing evidence that "seronegative" APS does exist and it may be that serological markers other than aPL and anti-B2GPI are important in these patients. Pathological livedo reticularis may therefore be a clinical marker of the "seronegative APS" [46,50].

## Conclusion

The spectrum of APS is not limited to thrombosis or pregnancy morbidity and clinicians should be aware of the broad range of manifestations with multi-system involvement.

## Competing interests

The authors declare that they have no competing interests.

## Authors' contributions

MM has written the manuscript, SRS has conceived and compiled the material and helped to write the manuscript and DPD has supervised the drafting of the manuscript. All authors have read and approved the final manuscript.

## References

[B1] Hughes GR (1983). Thrombosis, abortion, cerebral disease and the lupus anticoagulant. BMJ.

[B2] Miyakis S, Lockshin MD, Atsumi T, Branch DW, Brey RL, Cervera R, Derksen RH, DE Groot PG, Koike T, Meroni PL, Reber G, Shoenfeld Y, Tincani A, Vlachoyiannopoulos PG, Krilis SA (2006). International consensus statement on an update of the classification criteria for definite antiphospholipid syndrome (APS). J Thromb Haemost.

[B3] Khamashta MA, Cervera R, Asherson RA, Font J, Gil A, Coltart DJ, Vázquez JJ, Paré C, Ingelmo M, Oliver J (1990). Association of antibodies against phospholipids with heart valve disease in systemic lupus erythematosus. Lancet.

[B4] Christodoulou C, Sangle S, D'Cruz DP (2007). Vasculopathy and arterial stenotic lesions in the antiphospholipid syndrome. Rheumatology (Oxford).

[B5] Sangle SR, D'Cruz DP, Jan W, Karim MY, Khamashta MA, Abbs IC, Hughes GR (2003). Renal artery stenosis in the antiphospholipid (Hughes) syndrome and hypertension. Ann Rheum Dis.

[B6] Sangle SR, Jan W, Lau IS, Bennett AN, Hughes GRV, D'Cruz DP (2006). Coeliac artery stenosis and antiphospholipid (Hughes) syndrome/antiphospholipid antibodies. Clin Exp Rheumatol.

[B7] Wong M, Sangle S, Jan W, Hughes GR, D'Cruz DP (2005). Intracerebral arterial stenosis with neurological events associated with antiphospholipid syndrome. Rheumatology (Oxford).

[B8] Sipek-Dolnicar A, Hojnik J, Bozic B, Vizjak A, Rozman B, Ferluga D (2002). Clinical presentations and vascular histopathology in autopsied patients with systemic lupus erythematosus and anticardiolipin antibodies. Clin Exp Rheumatol.

[B9] Kojima E, Naito K, Iwai M, Hirose Y, Isobe K, Takano K (1997). Antiphospholipid syndrome complicated by thrombosis of the superior mesenteric artery, co-existence of smooth muscle hyperplasia. Intern Med.

[B10] Sangle SR, D'Cruz DP, Abbs IC, Khamashta MA, Hughes GR (2005). Renal artery stenosis in hypertensive patients with antiphospholipid (Hughes) syndrome: outcome following anticoagulation. Rheumatology (Oxford).

[B11] Rosenthal E, Sangle SR, Taylor P, Khamashta MA, Hughes GR, D'Cruz DP (2006). Treatment of mesenteric angina with antiphospholipid (Hughes) syndrome and coeliac artery stenosis. Ann Rheum Dis.

[B12] Remondino GI, Mysler E, Pissano MN, Furattini MC, Basta MC, Presas JL, Allievi A (2000). A reversible bilateral renal artery stenosis in association with antiphospholipid syndrome. Lupus.

[B13] Cervera R (2004). Coronary and valvular syndromes and antiphospholipid antibodies. Thromb Res.

[B14] Tenedios F, Erkan K, Lockshin MD (2005). Cardiac involvement in the antiphospholipid syndrome. Lupus.

[B15] Kaplan SD, Chartash EK, Pizzarello RA, Furie RS (1992). Cardiac manifestations of antiphospholipid syndrome. Am Heart J.

[B16] Vaarala O (1998). Antiphospholipid antibodies and myocardial infarction. Lupus.

[B17] Hamsten A, Norberg R, Bjorkholm M, de Faire U, Holm G (1986). Antibodies to cardiolipin in young survivors of myocardial infarctions: an association with recurrent cardiovascular events. Lancet.

[B18] Nair S, Khamashta MA, Hughes GRV (2002). Syndrome X and Hughes syndrome. Lupus.

[B19] Lagana B, Baratta L, Tubani L, Golluscio V, Delfino M, Rossi Fanelli F (2001). Myocardial infarction with normal coronary arteries in a patient with primary antiphospholipid syndrome. Case report and literature review. Angiology.

[B20] Davies JO, Hunt BJ (2007). Myocardial infarction in young patients without coronary atherosclerosis: assume primary antiphospholipid syndrome until proved otherwise. Int J Clin Pract.

[B21] Johnson BD, Shaw LJ, Pepine CJ, Reis SE, Kelsey SF, Sopko G, Rogers WJ, Mankad S, Sharaf BL, Bittner V, Bairey Merz CN (2006). Persistent chest pain predicts cardiovascular events in women without obstructive coronary artery disease: results from the NIH-NHLBI sponsored Women's ischaemia Syndrome Evaluation (WISE) study. Eur Heart J.

[B22] Kaski JC (2006). Cardiac syndrome X in women: the role of oestrogen deficiency. Heart.

[B23] Sangle S, D'Cruz D (2008). Syndrome X (angina pectoris with normal coronary arteries) and myocardial infarction in patients with anti-phospholipid (Hughes) syndrome. Lupus.

[B24] Kattwinkel N, Villanueva AG, Labib SB, Aretz HT, Walek JW, Burns DL, Klenz JT (1992). Myocardial infarction caused by microvasculopathy in a patient with primary antiphospholipid syndrome. Ann Int Med.

[B25] Sanna G, D'Cruz D, Cuadrado MJ (2006). Cerebral manifestations in the antiphospholipid (Hughes) syndrome. Rheum Dis Clin North Am.

[B26] Ciubotaru CR, Esfahani F, Benedict RH, Wild LM, Baer AN (2002). Chorea and rapidly progressive subcortical dementia in antiphospholipid syndrome. J Clin Rheumatol.

[B27] Tektonidou MG, Varsou N, Kotoulas G, Antoniou A, Moutsopolous HM (2006). Cognitive deficits in patients with antiphospholipid syndrome: association with clinical, laboratory, and brain magnetic resonance imaging findings. Arch Intern Med.

[B28] Cuadrado MJ, Khamashta MA, D'Cruz D, Hughes GRV (2001). Migraine in Hughes syndrome – heparin as a therapeutic trial?. QJM.

[B29] Tektonoidou MG, Malagari K, Vlachoyiannopoulos PG, Kelekis DA, Moutsopoulos HM (2003). Asymptomatic avascular necrosis in patients with primary antiphospholipid syndrome in the absence of corticosteroid use: a prospective study by magnetic resonance imaging. Arthritis Rheum.

[B30] Seleznick MJ, Silveira LH, Espinoza LR (1991). Avascular necrosis associated with anticardiolipin antibodies. J Rheumatol.

[B31] Egan RM, Munn RK (1994). Catastrophic antiphospholipid syndrome presenting with multiple thromboses and sites of avascular necrosis. J Rheumatol.

[B32] Sangle S, D'Cruz DP, Khamashta MA, Hughes GR (2004). Antiphospholipid antibodies, systemic lupus erythematosus, and non-traumatic metatarsal fractures. Ann Rheum Dis.

[B33] Vasoo S, Sangle S, Zain M, D'Cruz D, Hughes G (2005). Orthopaedic manifestations of the antiphospholipid (Hughes) syndrome. Lupus.

[B34] Davies RJ, Sangle SR, Khamashta MA, D'Cruz DP (2006). Antiphospholipid (Hughes) syndrome and atheroma. Lupus.

[B35] Brown MS, Goldstein JL (1983). Lipoprotein metabolism in the macrophage: implications for cholesterol deposition in atherosclerosis. A Rev Biochem.

[B36] Henriksen T, Mahoney EM, Steinburg D (1983). Enhanced macrophage degradation of biologically modified low density lipoprotein. Arteriosclerosis.

[B37] Barón MA, Khamashta MA, Hughes GR, D'Cruz DP (2005). Prevalence of an abnormal ankle-brachial index in patients with primary antiphospholipid syndrome: preliminary data. Ann Rheum Dis.

[B38] Christodoulou C, Zain M, Bertolaccini ML, Sangle S, Khamashta MA, Hughes GR, D'Cruz DP (2006). Prevalence of an abnormal ankle-brachial index in patients with antiphospholipid syndrome with pregnancy loss but without thrombosis: a controlled study. Ann Rheum Dis.

[B39] Charakida M, Halcox JP, Sangle S, D'Cruz D, Donald AE, Mackworth-Young CG, Klein NJ, Hughes G, Deanfield JE (2006). Endothelial Dysfunction and Increased Carotid Intima-Media Thickness in Primary Antiphospholipid Syndrome. Arthritis Rheum.

[B40] Medina G, Casaos D, Jara LJ, Vera-Lastra O, Fuentes M, Barile L, Salas M (2003). Increased carotid artery intima-media thickness may be associated with stroke in primary antiphospholipid syndrome. Ann Rheum Dis.

[B41] Margarita A, Batuca J, Scenna G, Alves JD, Lopez L, Iannaccone L, Matsuura E, Ames PR (2007). Subclinical atherosclerosis in primary antiphospholipid syndrome. Ann N Y Acad Sci.

[B42] Chaib AI, Mellilo N, Sangle SR, Sabharwal T, Tungekar F, Abbs IC (2007). Antiphospholipid antibodies and increased bleeding complications following renal biopsy: a single centre study. Arthritis Rheum.

[B43] Roubey RAS, Roch B, Amengual O, Atsumi T, Khamashta MA, Hughes GRV, Khamashta MA (2000). Antiphospholipid antibody-negative syndrome – other phospholipids. Hughes Syndrome Antiphospholipid syndrome.

[B44] Carmo-Pereira S, Bertolaccini ML, Escudero-Conteras A, Khamashta MA, Hughes GR (2003). Value of IgA anticardiolipin and anti-beta2-glycoprotein I antibody testing in patients with pregnancy morbidity. Ann Rheum Dis.

[B45] Frances C, Niang S, Laffitte E, Pelletier F, Costedoat N, Piette JC (2005). Dermatologic manifestations of the antiphospholipid syndrome: two hundred consecutive cases. Arthritis Rheum.

[B46] Sangle S, Christodoulou C, Paul S, Hughes GR, D'Cruz DP (2008). The point prevalence of an abnormal ankle-brachial index in antiphospholipid antibody negative patients with livedo reticularis: a controlled study. Ann Rheum Dis.

[B47] Frances C, Papo T, Wechsler B, Laporte JL, Biousse V, Piette JC (1999). Sneddon Syndrome with or without antiphospholipid antibodies, A comparative study in 46 patients. Medicine (Baltimore).

[B48] Weinstein S, Piette W (2008). Cutaneous manifestations of antiphospholipid antibody syndrome. Hematol Oncol Clin North Am.

[B49] Kriseman YL, Nash JW, Hsu S (2007). Criteria for the diagnosis of antiphospholipid syndrome in patients presenting with dermatologic symptoms. J Am Acad Dermatol.

[B50] Hughes GRV, Khamashta MA (2003). Seronegative antiphospholipid syndrome. Ann Rheum Dis.

